# Supplementary material for: Participant-derived cell line transcriptomic analyses and mouse studies reveal a role for ZNF335 in plasma cholesterol statin response
Source: Genome Med. 2024 Jul 26;16:93. doi: 10.1186/s13073-024-01366-9 (PMC11282643; doi:10.1186/s13073-024-01366-9)
Supplement: Supplementary file 2 — Additional file 2: Supplementary figures. Fig. S1 Correlation of pre-statin plasma HDL-cholesterol with ZNF335 LCL gene expression levels from the corresponding donors split by sex (N = 211 men, N = 216 women). Fig. S2 Mouse growth curves split by Zfp335 genotype and sex. Adjusted p values were calculated using the Compare Groups of Growth Curves (CGGC) method with 10,000 permutation tests. Male sample sizes were N = 7, 19, and 7 and female sample sizes were N = 7, 8, and 4 for wild-type, heterozygotes, and homozygous Zfp335R1092W, respectively. Values are mean ± SD. *p < 0.05, **p < 0.01. Fig. S3 Statin-induced changes in wild-type N = 5 male and N = 11 female mouse lipoprotein profiles measured by ion mobility. Mouse profiles were measured before and after 4 weeks of simvastatin-containing diet. Fig. S4 Statin-induced changes in (A) male and (B) female mouse plasma lipoprotein composition split by Zfp335 genotype as measured by ion mobility. Male sample sizes were N = 5, 12, and 5 and female sample sizes were N = 11, 11, and 5 for wild-type, heterozygotes, and homozygous Zfp335R1092W, respectively. The size intervals designating the major lipoprotein subclasses are based on those defined in humans [29]. Values are mean ± SD. Fig. S5 Ion mobility lipoprotein profiles for N = 803 CAP participants before and after 6 weeks of 40 mg/day simvastatin treatment. Profiles shown are (A) pre-statin split by sex and race/ethnicity, (B) on-statin split by sex and race/ethnicity, (C) pre- and on-statin for all, and (D) pre-statin and on-statin split by sex. N = 142 for female African American, N = 142 for male African American, N = 243 for female white, and N = 276 for male white participants. Fig. S6 Correlation of plasma LDL-cholesterol statin response with ZNF335 LCL gene expression statin response from the corresponding donors split by sex (N = 211 men, N = 216 women) [file 13073_2024_1366_MOESM2_ESM.pdf]

Supplementary Figures

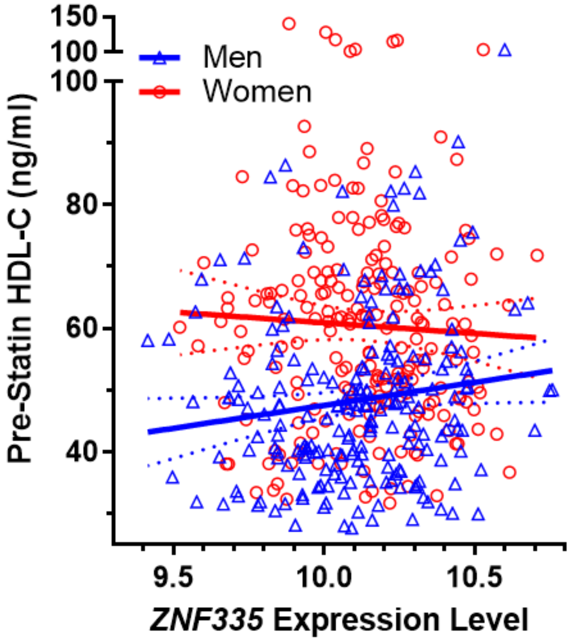

Fig. S1. Correlation of pre-statin plasma HDL-cholesterol with *ZNF335* LCL gene expression levels from the corresponding donors split by sex (N=211 men, N=216 women).

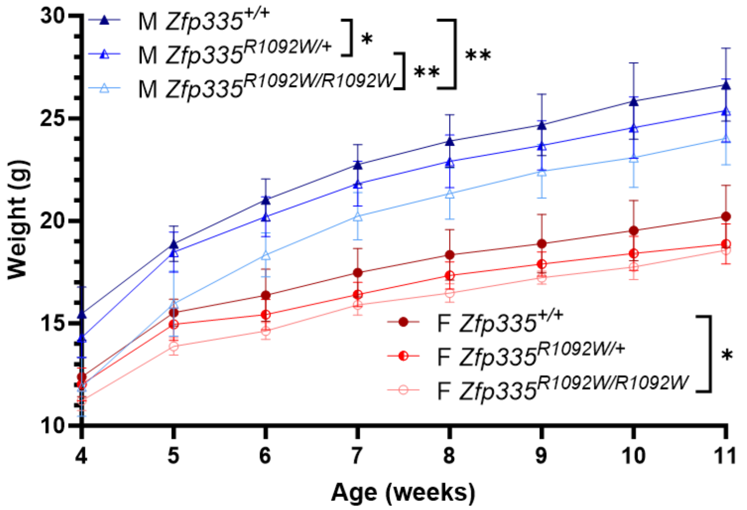

Fig. S2. Mouse growth curves split by *Zfp335* genotype and sex. Adjusted p-values were calculated using the Compare Groups of Growth Curves (CGGC) method with 10,000 permutation tests. Male sample sizes were N=7, 19, and 7 and female sample sizes were N=7, 8, and 4 for wild type, heterozygotes, and homozygous $Zfp335^{R1092W}$, respectively. Values are mean ± SD. *p<0.05 **p<0.01

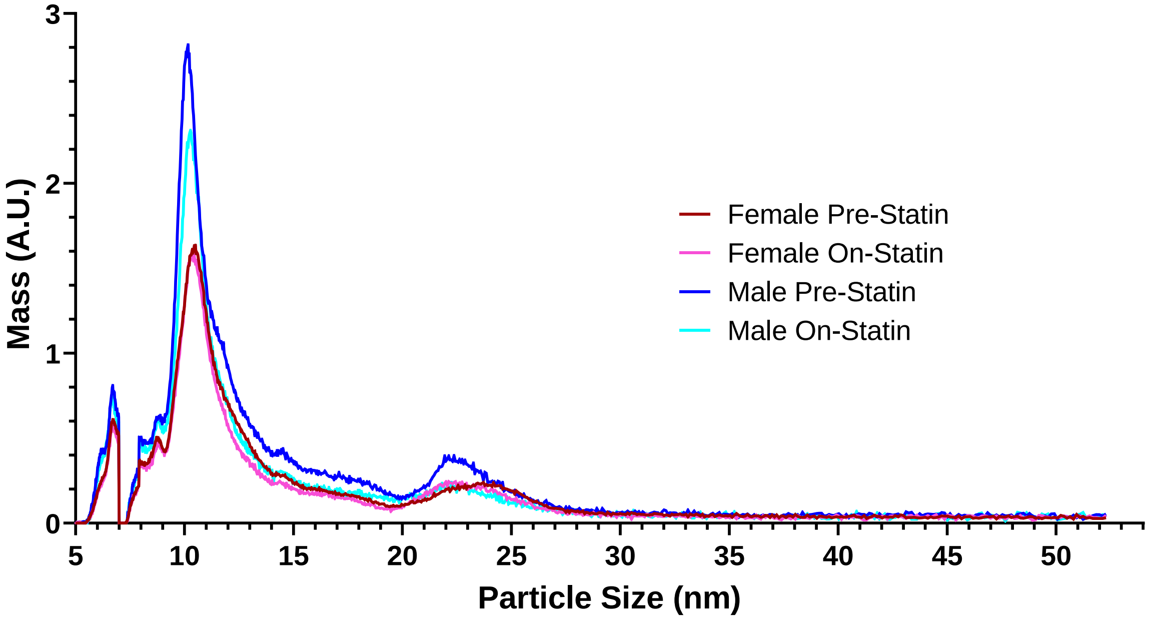

Fig. S3. Statin-induced changes in wild-type N=5 male and N=11 female mouse lipoprotein profiles measured by ion mobility. Mouse profiles were measured before and after 4 weeks of simvastatin-containing diet.

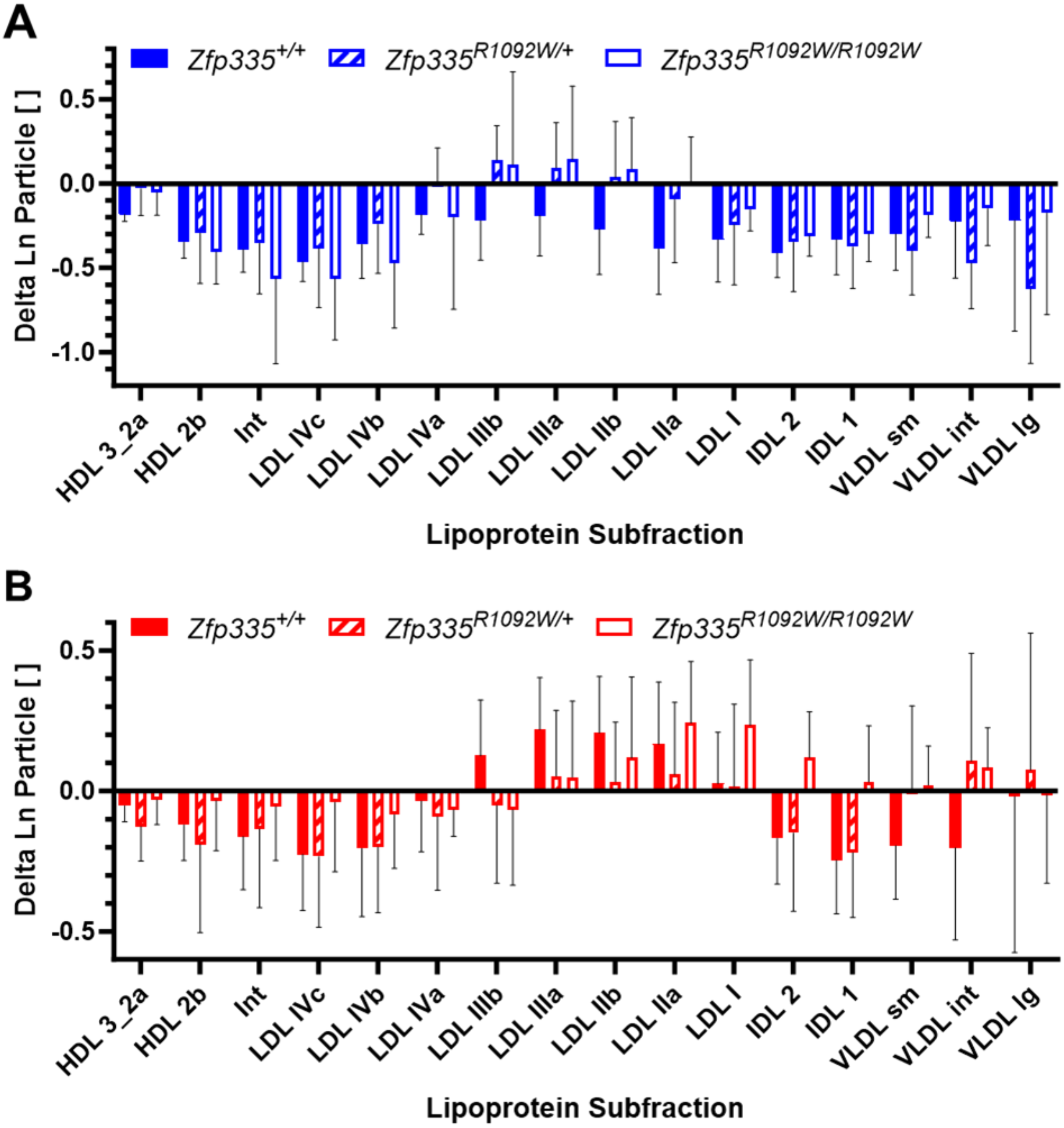

Fig. S4. Statin-induced changes in (A) male and (B) female mouse plasma lipoprotein composition split by *Zfp335* genotype as measured by ion mobility. Male sample sizes were N=5, 12, and 5 and female sample sizes were N=11, 11, and 5 for wild type, heterozygotes, and homozygous Zfp335^{R1092W}, respectively. The size intervals designating the major lipoprotein subclasses are based on those defined in humans (29). Values are mean ± SD.

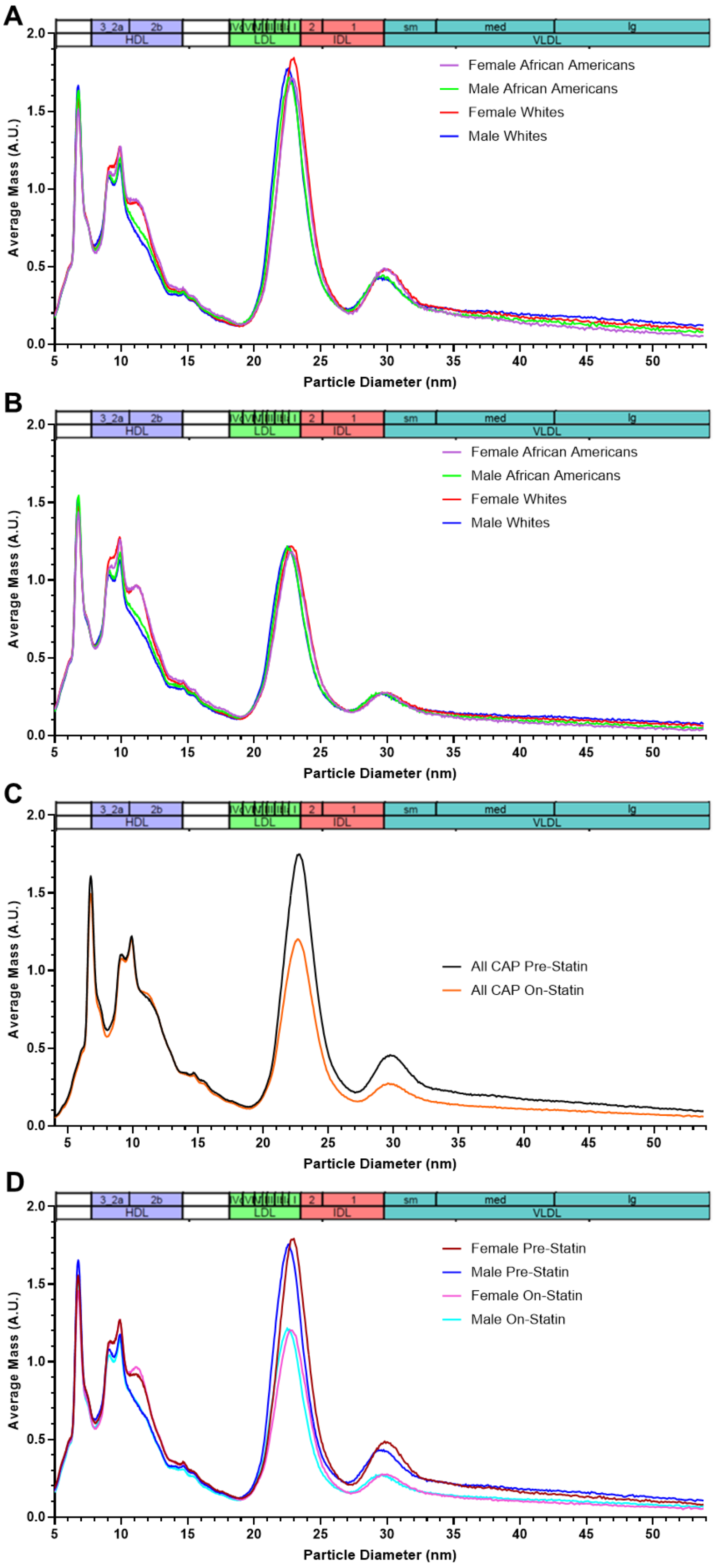

Fig. S5. Ion mobility lipoprotein profiles for N=803 CAP participants before and after 6 weeks of 40 mg/day simvastatin treatment. Profiles shown are A) pre-statin split by sex and race/ethnicity B) on-statin split by sex and race/ethnicity C) pre- and on-statin for all and D) pre-statin and on-statin split by sex. N=142 for female African American, N=142 for male African American, N=243 for female white, and N=276 for male white participants.

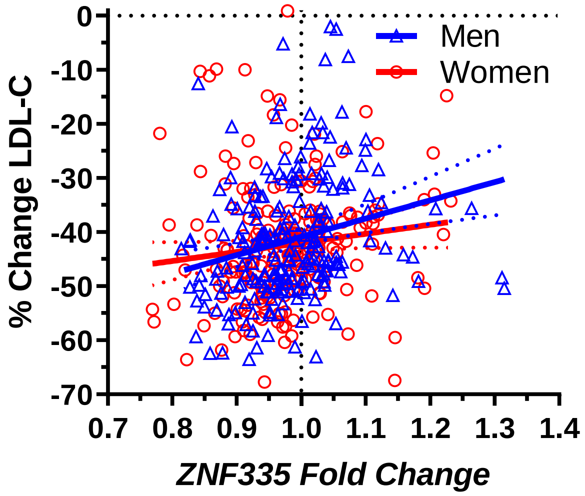

Fig. S6. Correlation of plasma LDL-cholesterol statin response with *ZNF335* LCL gene expression statin response from the corresponding donors split by sex (N=211 men, N=216 women).
